# Clinical Relevance of Plasma Concentrations of MBL in Accordance with IgE Levels in Children Diagnosed with Bronchial Asthma

**DOI:** 10.3390/medicina56110594

**Published:** 2020-11-06

**Authors:** Simona Maria Borta, Simona Dumitra, Imola Miklos, Romana Popetiu, Luminița Pilat, Maria Pușchiță, Cătălin Marian

**Affiliations:** 1 Department of Internal Medicine Clinic I, Faculty of Medicine, “VasileGoldiș” Western University of Arad, 310045 Arad, Romania; popetiur@yahoo.ca (R.P.); mpuschita@gmail.com (M.P.); 2 Department of Pediatric Clinic II, Faculty of Medicine, “VasileGoldiș” Western University of Arad, 310045 Arad, Romania; dumitrasimona@yahoo.com; 3 Department of Physiology, Faculty of Medicine, “VasileGoldiș” Western University of Arad, 310045 Arad, Romania; miklosimola@gmail.com; 4 Department of Biochemistry, Faculty of Medicine, “Vasile Goldiș” Western Universtiy of Arad, 310045 Arad, Romania; luminita.pilat@yahoo.com; 5 Department of Biochemistry, Faculty of Medicine, University of Medicine and Pharmacy “Victor Babes”, 300041 Timișoara, Romania; cmarian@umft.ro

**Keywords:** bronchial asthma, children, mannose binding lectin, immunoglobulin E, allergies, eosinophilic cells

## Abstract

*Background and objectives:* Bronchial asthma is a heterogeneous, multifactorial pulmonary disease characterized by variable airway obstruction caused by chronic inflammation. Our study investigates the clinical relevance of MBL plasma levels in accordance with IgE values in children who attended a pediatric consult for respiratory symptoms with bronchial asthma. *Materials and Methods:* The study population consists of patients <18-years-old and included 43 patients with bronchial asthma and 64 age-matched healthy subjects as a control group. We used the ELISA Human MBL Immunoassay kit and the electrochemiluminescence immunoassay (ECLIA) kit for IgE determination. *Results:* Our results show significantly different distributions of patients in the bronchial asthma group and control group. The measured values were within the normal range for most controls, while the bronchial asthma patients displayed higher values of plasma MBL and IgE levels. We observed a wider heterogeneity in MBL concentrations in bronchial asthma patients when compared to the healthy age-matched controls. Our results also suggest a potential clinical usefulness of plasma MBL concentrations in accordance with IgE and eosinophil cells levels in the diagnosis of bronchial asthma, and our results may suggest a prognostic role of MBL in the evolution of asthmatic disease; however, further studies are necessary to confirm these findings. *Conclusions:* We can say that plasma MBL concentrations present a relative diagnostic role for bronchial asthma in pediatric patients and may suggest a more severe disease progression; however, further studies are needed to elucidate the role played by MBL in the determination and evolution of this disease.

## 1. Introduction

Bronchial asthma is a heterogeneous, multifactorial pulmonary disease characterized by variable airway obstruction caused by chronic inflammation [1], related to the interaction between the respiratory epithelium, the innate immune system, and the adaptive immunity [2,3]. The respiratory epithelium is exposed to numerous stimuli, such as allergens, infectious agents, pollution, and oxidants, and is able to produce a number of mediators that can initiate the immune system [4].

The most important immune factors in the pathogenesis of bronchial asthma are the eosinophil cells and the immunoglobulin E (IgE). Although there is currently a wealth of data on these factors, most studies address the role played by the mannose binding lectin (MBL) gene and the homonymous protein in the pathogenesis of bronchial asthma [5]. However, the results of these studies are still contradictory, with some works pointing to the important role of MBL in the pathogenesis of bronchial asthma. Eosinophilic cells, which are abundant the airways in most patients with bronchial asthma, are stimulated by factors released from the airway epithelial cells (Th2 cells and mast cells) and express a wide variety of pro-inflammatory cytokines [6]. Another key player in this pathology, IgE, is an antibody secreted by circulating B cells in response to bacteria, viruses, and other microorganisms, as well as to substances recognized as non-self or antigens to the immune system. Itis maintained on the surfaces of mast cells and basophils even in the absence of the allergen. Upon subsequent encounter with the same allergens, the antigen binds to IgE, stimulating the release of pro-inflammatory mediators, such as histamine, leukotrienes, and cytokines [7]. Binding of the allergen to the IgE on the cell surface induces the signal transduction cascade [8,9].

Mannose binding lectin is a 32 kDa polypeptide encoded by the *Mbl-2* gene on chromosome 10 (10q11.2–q21) that plays an essential role in innate immunity [10]. MBL recognizes the molecular patterns associated with pathogens and determines the lysis of microorganisms by activating the complement in the lectin pathway. In addition, MBL intensifies the phagocytosis process by direct opsonization involving MBL receptors from the phagocyte surface [11,12]. A low level of circulating MBL has been observed in children with increased susceptibility to respiratory infections, but data on the role of MBL in bronchial asthma are contradictory. Thus, different levels of circulating MBL have been found in the blood of patients with bronchial asthma, due to the heterogeneity of the disease [5]. In this context, our study aimed to investigate the clinical relevance of MBL plasma levels in accordance with IgE values in children who attended a pediatric consult for respiratory symptoms with bronchial asthma.

## 2. Materials and Methods

### 2.1. Study Population

This is a prospective study that aims to analyze the correlations between the IgE levels and serum MBL levels in pediatric patients with bronchial asthma. The study population consists of patients <18-years-old whose parents provided written consent to participate; we also obtained assent from teenage participants who fulfilled the inclusion criteria (pediatric patients <18 years, who presented forced expiratory volume (FEV) variability and were under treatment with bronchodilators and antihistamines). The study was conducted in accordance with the ethical principles from the Declaration of Helsinki, which respects ethical demands that are required for research with human subjects. Before starting the study, approval from the Human Research and Ethics Committee of the “VasileGoldiș” Western University of Arad and from the Emergency County Hospital, Arad, Romania, was obtained (number of approval 2/19.07.2018 and 128/7.12.2018). The study was carried out at the Pediatric Clinic, Emergency County Hospital, Arad, Romania, during a 1-year period between 2019 and 2020, and included 43 patients with bronchial asthma who attended a pediatric consult for respiratory symptoms and 64 age-, sex-, and race-matched healthy subjects as a control group. All patients were interviewed for personal medical history, allergies, family history, and parent’s toxic substances consumption (alcohol, smoking, etc.). In addition, ventilatory probes were performed in order to evaluate the pulmonary functions and blood samples were collected to determine the IgE quantitative levels and the MBL plasma concentrations. The purpose of our study was to evaluate the clinical relevance of plasma MBL concentrations in concordance with IgE levels in bronchial asthma diagnosis in patients <18 years. The IgE levels and serum MBL concentrations of asthmatic children were measured during the remission periods.

### 2.2. Quantification of Plasma MBL and IgE Levels

For the quantitative determination of plasma MBL levels, we used the Quantikine ELISA Human MBL Immunoassay kit according to the manufacturer’s instructions (R&D Systems). For the multichannel absorbance reading of ELISAs, we utilized the Tecan’s Sunrise absorbance microplate reader, with a temperature control function for enzyme kinetic assays. To determine IgE levels, we used the electrochemiluminescence immunoassay (ECLIA) kit on a COBAS E 601 immunoassay analyzer, according to the manufacturer’s recommendations (La Roche Laboratories). All assays were done in a certified clinical laboratory setting.

### 2.3. Statistical Analysis

The statistical analyses were conducted using Microsoft Office Excel 2016 and Statistica 7 (StatSoft Inc., Tulsa, OK, USA), considering a *p* value< 0.05 for statistical significance. First, the subjects were stratified based on sex and health status into healthy girls, healthy boys, asthmatic girls, and asthmatic boys. Before applying ANOVA, the corresponding age-related datasets were verified for normality with Anderson–Darling tests and for homoscedasticity (homogeneity of variances) with a Bartlett test. A similar approach was used for log-transformed data (decimal logarithm) for IgE levels and MBL levels. In the case of significant results, posthoc analysis was run using Tukey’s *HSD* tests for unequal samples. We also examined the relationships between the variables for each category of patients using Pearson’s correlations. Finally, we analyzed the differences in IgE levels and MBL levels among heathy subjects and asthmatic subjects using t tests for unequal sample sizes, as well as the correlations between these outcomes per each category of subjects. Prior to these analyses, the datasets with log-transformed data (decimal logarithm) were checked for normality and homoscedasticity with Anderson–Darling tests and with F tests, respectively.

## 3. Results

Regarding the comorbidities of the patients enrolled in this study, we found that out of the total number of subjects with bronchial asthma (n = 43), 7.24% were diagnosed with allergic-type comorbidities and 2.89% had intellectual disability; the rest of the patients did not present risk factors or comorbidities. At admission, all patients in the group of asthmatics showed FEV1 variability. Of the total asthma patients, 17 had one parent diagnosed with bronchial asthma or other allergic pathologies, and only one had both parents diagnosed with this pathology.

**Figure 1:**
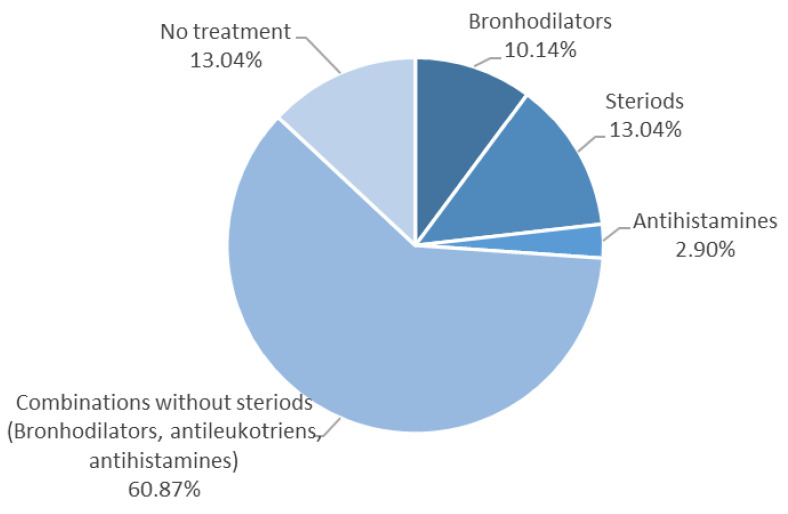
Treatment given to patients before sampling.

Figure 1 shows the treatment received by patients at the time of blood sampling. It can be observed that 13.04% of bronchial asthma patients had no treatment, 13.04% received steroids, 10.14% were under bronchodilators, 2.9% received antihistamines, whereas most subjects (60.87%) were given a combined treatment with bronchodilators, antileukotrienes, and antihistamines, without steroids. In the study, only inhaled steroids were used, that is 100 mcg of fluticasone or 80 mcg budesonide.

In the study patients, the overall median values (with lower and upper quartiles) for age, count of eosinophilic cells, IgE levels, and MBL levels were: 8.09 years (2, 17), 8.8% (4.7, 12.4), 367.07 UI/mL (0.617, 2500), and 5.37 ng/mL (0.406, 16.159), respectively. In this study, we did not include infants because they may not manifest a big enough immune response.

The data for age and the log_10_-transformed data for IgE levels and MBL levels were normally distributed (Anderson–Darling test, *p* ≥ 0.105). The measured datasets were also homoscedastic for all analyzed variables, i.e., age (Bartlett’s test, *p* = 0.054), IgE levels (Bartlett’s test, *p* = 0.291), and MBL levels (Bartlett’s test, *p* = 0.273). These results reveal that the requirements underlying the application of the ANOVA were fulfilled for all three variables.

**Figure 2:**
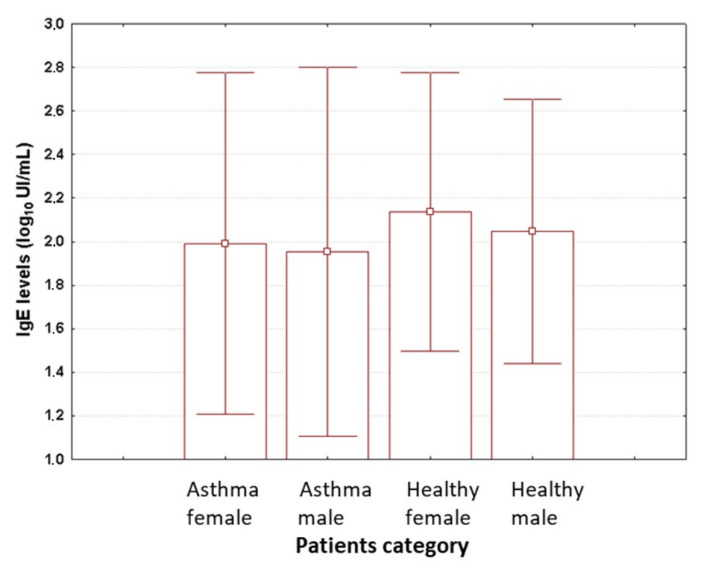
The measured values for IgE levels when patients were stratified based on sex and health status. Data are shown on a log_10_ scale as mean (box) and one standard deviation (error bar). Asthma female—asthmatic girls. Asthma male—asthmatic boys. Healthy female—healthy girls. Healthy male—healthy boys.

The age data were similar across different categories of patients (ANOVA, *p* = 0.104). This confirms the homogeneity of groups with respect to patient’s age. Table 1 displays the average values for age across different study groups when stratified by gender and health status. The mean levels of IgE, as shown in Figure 2, showed the highest values for healthy girls (536.68 UI/mL), followed by asthmatic boys (338.98 UI/mL), asthmatic girls (311.62 UI/mL),and healthy boys (245.49 UI/mL). However, these differences did not reach statistical significance (ANOVA, *p* = 0.664)—like in the case of age.

**Figure 3:**
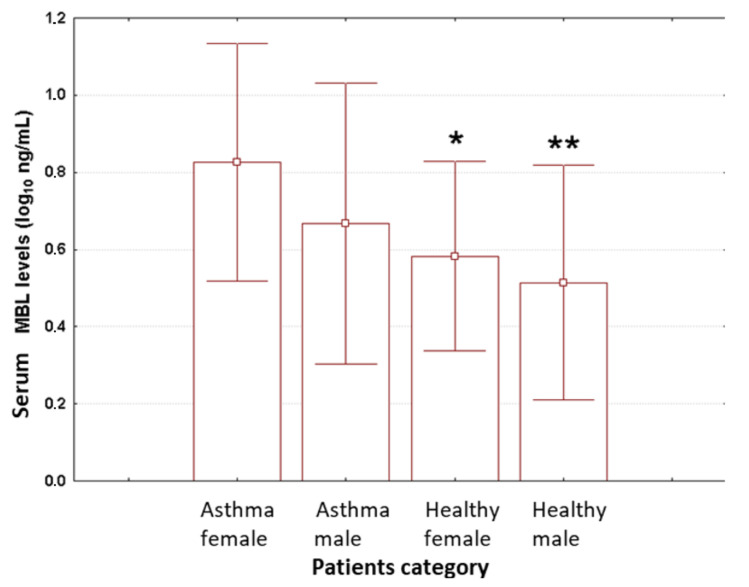
The measured values for serum MBL levels when patients were stratified based on sex and health status. Data are shown on a log_10_ scale as mean (box) and one standard deviation (error bar). Marked boxes (*) indicate significant differences as compared to the first group from the left (Tukey’s *HSD test* for *unequal samples*, **—*p* < 0.01, *—*p* < 0.05). Asthma female—asthmatic girls. Asthma male—asthmatic boys. Healthy female—healthy girls. Healthy male—healthy boys.

In contrast, we identified significant differences in plasma MBL levels when subjects were stratified based on gender and health status (ANOVA, *p* < 0.001). The measured average values for serum MBL were the greatest for asthmatic girls (8.15 ng/mL), followed by asthmatic boys (5.97 ng/mL), healthy girls (4.36 ng/mL), and healthy boys (3.99 ng/mL), as shown in Figure 3. Post hoc analysis revealed significant differences in the measured values for this parameter between asthmatic girls and either healthy girls or healthy boys (Tukey’s *HSD test* for *unequal samples*; *p* = 0.035 and *p* = 0.003, respectively). The other post hoc comparisons yielded non-significant results (Tukey’s *HSD tests* for *unequal samples, p ≥ 0.343*). We also found no significant correlations between IgE levels and MBL levels, irrespective of analyzed group (*p* ≥ 0.219).

When the patients were grouped based on their health status in healthy patients and asthmatic patients, the log_10_-transformed data for IgE levels and MBL levels were found to be normally distributed (Anderson–Darling test, *p* ≥ 0.143) and homoscedastic (F test, *p* ≥ 0.063). The obtained results showed a similar trend with that observed when the subjects were stratified based on both sex and health status. Thus, the application of t tests revealed significant differences in the case of plasma MBL, with the measured average values being significantly greater in the case of asthmatic subjects as compared to healthy subjects (7.13 vs. 4.18; *t* test, *p* = 0.026). In contrast, no significant results were detected for IgE (395.64 vs. 324.54; *t* test, *p* = 0.429). Moreover, no significant relationships were identified between IgE levels and plasma MBL levels, in both healthy and asthmatic patients *(p* ≥ 0.119).

## 4. Discussion

To our knowledge, this is the first study to examine in Romania the clinical relevance of plasma MBL levels in the context of bronchial asthma in patients under 18 years. Our research’s results should therefore expand previous knowledge about the putative link between MBL and pathogenesis of bronchial asthma in children and offer new data regarding new potential tools to be used for simplifying and improving the diagnostic of this pathology pediatric patients.

Our study focused on the determination of plasma IgE levels in patients stratified based on the levels of MBL and eosinophilic blood cells in systemic bloodstream. We found 12 patients with bronchial asthma who showed elevated levels of eosinophil cells in the blood samples, while the subjects from the control group revealed normal eosinophil counts. This suggests that eosinophil count may be useful for delineating between children with bronchial asthma and healthy children.

Once present in the airway and systemic blood circulation, the eosinophil cells release some substances, which will determine bronchoconstriction and deterioration changes of the epithelial cells. This damage, related to the profibrotic cytokines also released by eosinophils and epithelial cells, may lead to airway remodeling [13].

In various pathological conditions, the activation of the MBL-mediated complement pathway was demonstrated. Thus, the role of increased MBL levels and complement activity in chronic renal failure, diabetic nephropathy, and rheumatic heart disease was reported [14].

Mannan-binding lectin has been considered an acute phase reactant and its plasmatic concentrations grow by up to threefold in response to different stimuli such as infectious agents or surgery [15]. Some studies have found elevated plasmatic MBL concentrations and high activity in allergic patients. In allergic patients, besides the increased MBL plasmatic levels, there is an increase in eosinophil cells levels, which remain high during the remission periods of the disease [16,17].

Elevated levels of MBL and its activity in allergic patients may contribute to additional activation of complement by the lectin pathway and therefore, to an increased severity of allergic markers, such as an increase in the number of eosinophils in the blood. Complementary activation of MBL can also lead directly to the development of allergies by affecting the levels of proinflammatory cytokines [18].

Therefore, the study herein aimed to contribute to the discovery of a possible minimally invasive diagnostic or prognostic test based on the measurement of serum MBL levels in accordance with the level of IgE and eosinophilic cells in allergic bronchial asthma from early age.

Here, we used a quantitative, sandwich ELISA procedure to investigate differences in the distribution of serum MBL levels among the patients from the two study groups. The overall range of serum was comparable to that reported in a previous research work using the same ELISA-based approach for the quantification of serum MBL levels in children of comparable age and health status. The measured values were between 3.6 and 4.1 mg/L in pediatric patients with bronchial asthma, and similar to our investigation, they were higher than the values seen in healthy patients [17].

Another study reported a mean serum MBL of 686.7 ± 339.2 ng/mL in adult bakery workers suffering from bronchial asthma, which was significantly higher than the average level determined for the control group (358.3 ± 180.4 ng/mL). The measured values in patients with chronic obstructive pulmonary disease were even higher, reaching 918 ng/mL [19].

In our study, plasma MBL concentrations were significantly higher in children with bronchial asthma compared to the healthy controls. We also found a positive correlation between plasma MBL concentrations and IgE levels in peripheral blood samples. These data provide evidence for an association between these two biomarkers, which may reflect similar molecular aspects underlying disturbed airway functions. However, this association is difficult to be taken into account as an indication of MBL’s role in the pathogenesis of allergic asthma in pediatric patients, given the fact that correlation does not imply causation. Rather, it should be considered as an interesting finding which deserves further investigation.

Our study has its limitations. One such limitation is represented by the fact that the samples were collected during the remission periods in children already diagnosed with bronchial asthma. The second limitation is due to the fact that the plasmatic MBL levels may vary slightly among individuals depending on the environmental factors (e.g., arsenic, lead, persistent organic pollutants, air pollution) and infections or other inflammatory pathologies, which could possibly interfere with the obtained results [20].

However, our results are promising. We believe that the determination of plasma MBL in the future can be used as a diagnostic and prognostic test in bronchial asthma.

These findings support previous research studies that have reported increased levels of MBL in both adults and children diagnosed with bronchial asthma. Studies suggest that MBL might play a modulatory role in the progression of asthma and thus, be a marker of susceptibility to more severe disease or a marker for a distinctive asthma phenotype. Two authors reported an association between MBL levels and eosinophilia, supporting our hypothesis of a role for MBL in different asthma phenotypes [5].

## 5. Conclusions

Our study investigated for the first time the potential clinical significance of plasma MBL concentrations in the context of bronchial asthma in children. The measured values showed a wider heterogeneity in patients with bronchial asthma than in controls. Our results show that the plasma MBL concentrations compared to IgE values present a relative diagnostic role for bronchial asthma in pediatric patients, because MBL concentrations have higher relevance in the diagnosis of bronchial asthma in children and higher sensitivity and specificity, given that all asthmatic patients had altered MBL values compared to IgE values, which were modified in a few patients with asthma. The discrepancy between IgE values among the study groups requires further studies; further studies are needed as well to elucidate the role played by MBL in the determinism and evolution of bronchial asthma.

## Figures and Tables

**Table 1:** The measured values of count of eosinophilic cells stratified by gender and health status.

Study Population	Patients (no.)	Age (yrs.)	Count of Eosinophilic Cells (%)
Bronchial asthma Patients			8.8 (4.7, 12.4)
**Female**	23	9.34 ± 5.11	-
**Male**	20	7.9 ± 5.14	-
Control group			<7
**Female**	33	9.54 ± 5.20	-
**Male**	31	7.03 ± 4.11	-

Legend: Data related to age and count of eosinophilic cells for different groups of patients are shown as mean values with one standard deviation.
